# Structural Characterization of Natural Yeast Phosphatidylcholine and Bacterial Phosphatidylglycerol Lipid Multilayers by Neutron Diffraction

**DOI:** 10.3389/fchem.2021.628186

**Published:** 2021-03-18

**Authors:** Alessandra Luchini, Giacomo Corucci, Krishna Chaithanya Batchu, Valerie Laux, Michael Haertlein, Viviana Cristiglio, Giovanna Fragneto

**Affiliations:** ^1^ Niels Bohr Institute, University of Copenhagen, Copenhagen, Denmark; ^2^ Institut Laue Langevin, Grenoble, France; ^3^ Université Grenoble Alpes, Ecole Doctorale de Physique, Saint-Martin-d’Héres, France

**Keywords:** natural lipids, lipid multilayers, neutron diffraction, Pichia pastoris, *Escherichia coli*, deuterated lipids

## Abstract

Eukaryotic and prokaryotic cell membranes are difficult to characterize directly with biophysical methods. Membrane model systems, that include fewer molecular species, are therefore often used to reproduce their fundamental chemical and physical properties. In this context, natural lipid mixtures directly extracted from cells are a valuable resource to produce advanced models of biological membranes for biophysical investigations and for the development of drug testing platforms. In this study we focused on single phospholipid classes, i.e. *Pichia pastoris* phosphatidylcholine (PC) and *Escherichia coli* phosphatidylglycerol (PG) lipids. These lipids were characterized by a different distribution of their respective acyl chain lengths and number of unsaturations. We produced both hydrogenous and deuterated lipid mixtures. Neutron diffraction experiments at different relative humidities were performed to characterize multilayers from these lipids and investigate the impact of the acyl chain composition on the structural organization. The novelty of this work resides in the use of natural extracts with a single class head-group and a mixture of chain compositions coming from yeast or bacterial cells. The characterization of the PC and PG multilayers showed that, as a consequence of the heterogeneity of their acyl chain composition, different lamellar phases are formed.

## Introduction

In cell membranes, lipids are organized in a bilayer-like structure with their hydrophobic acyl chains pointing towards each other and the polar headgroups exposed to the extracellular and cytoplasmic aqueous media. Whereas both eukaryotic and prokaryotic cells are delimited by a cytoplasmic membrane, their lipid composition is significantly different (van Meer and Voelker, 2008; Harayama and Riezman, 2018). In the eukaryotic plasma membrane, phospholipids and sterols are the main membrane components (Maxfiled and van Meer, 2010), and among the phospholipids, phosphatidylcholine (PC) is the most abundant (Harayama and Riezman, 2018). Among the prokaryotic cells, most of the bacteria have only phospholipids as lipid components of their cytoplasmic membrane (Strahl and Errington, 2017) and more specifically phosphatidylethanolamine (PE) and phosphatidylglycerol (PG) are the most abundant (Romantsov and Wood, 2016).

The direct investigation of cell membranes has been limited by their high level of complexity, which makes their isolation difficult and prevents the implementation of the most common biophysical methods (Seddon et al., 2004). For this reason, most of the information available on the structure, dynamics and function of biological membranes have been obtained so far by using model systems that are characterised by a considerably simpler lipid composition, typically 1–3 synthetic phospholipid species, and, therefore, are easy to produce (Bagatolli et al., 2010; Mouritsen, 2011). In particular, liposomes, supported lipid bilayers, lipid monolayers and lipid multilayers are among the most common model systems for studying lipid membranes in solution or at solid/liquid and liquid/air interfaces (Katsaras and Gutberlet, 2001; Hardy et al., 2013). They are also often used as platforms to investigate the structure and function of membrane proteins (Retel et al., 2017; Luchini et al., 2019) and to test the activity of drugs (Seddon et al., 2009; Vitiello et al., 2015). These applications prompted the design of more advanced model membrane systems that better resemble the lipid composition of real cell membranes, while still preserving the compatibility with biophysical methods for their structural and dynamical characterization. In particular, the extraction of lipids from eukaryotic or bacterial cells, resulted to be a successful strategy to produce membrane systems closer to real cell membranes compared to the ones composed by the simpler synthetic lipid mixtures (Lind et al., 2015; Himbert et al., 2017; Fragneto et al., 2018).

In this context, we recently reported the production and characterization of natural membranes composed of lipid mixtures extracted from the yeast *Pichia pastoris* (de Ghellinck et al., 2014; Gerelli et al., 2014; de Ghellinck et al., 2015; Luchini et al., 2018; Luchini et al., 2020). The study of these lipid mixtures is relevant for the general understanding of eukaryotic membranes as well as for the development of lipid platforms to investigate the activity of antifungal drugs (de Ghellinck et al., 2015). More specifically, in one of our previous studies we investigated the structure of lipid multilayers composed of the total *P.pastoris* lipid extract by means neutron diffraction measurements (Luchini et al., 2018). The *P.pastoris* total lipid extract is a complex lipid mixture, including different phospholipid species, *i.e.* different headgroup and acyl chain composition, as well as sterols, *i.e.* ergosterol, steryl-esters, free fatty acids and triglycerides. We compared the structure of the multilayers prepared with the total lipid extract with that of multilayers prepared with only the phospholipid extract from *P.pastoris* (Luchini et al., 2018). The phospholipid extract still contains several phospholipid species with different headgroup and acyl chain composition. The collected neutron diffraction data showed self-segregation of the lipids in regions of the multilayers with different d-spacing, *i.e.* the characteristic repetitive distance within the multilayer. This observation suggested the coexistance of different lipid phases in both the multilayer prepared with the total lipid extract and the phospholipid extract. However, being these lipid extracts characterized by both an heterogeneous composition of the lipid headgroups and acyl chains, we were unable to identify if both the headgroups and the acyl chains could induce the observed lipid self-segregation. In these previous studies, neutron diffraction experiments were performed on lipid multilayers prepared with both hydrogenous and deuterated lipid extracts. Indeed, we previously described the production of natural deuterated lipids by growing *P.pastoris* cells in a deuterated culture media (de Ghellinck et al., 2014). Deuterated lipids are valuable for the investigation of biological membranes with neutron scattering methods but also NMR and infrared spectroscopy. Indeed, deuterated lipids can be used in mixtures with hydrogenous compounds and exploit the contrast variation method that allows highlighting the parts of interest of the system under study. They can also be used instead of hydrogenous lipids to improve signal to noise ratio.

In the present study, we continued our characterization of lipid extracts from *P. pastoris* and more specifically we focused on phosphatidylcholine (PC) lipid extracts. PC lipids are the most abundant lipids in *P.pastoris* as well as in mammalian membranes. The structure of multilayers prepared with these PC extracts are presented here for the first time. The interest of the study is that such extracts have a complex composition in terms of acyl chains (Table 1 and Supplementary Material) which enabled us to investigate the impact of the heterogeneous acyl chain composition on the lipid self-segregation within a multilayer. Neutron diffraction was used also in the present study as characterization method for the investigation of multilayers prepared with with hydrogenous or deuterated PC lipid extracts. Drop casting of lipid solutions both in organic solvents or water are the most common protocols for the preparation of lipid multilayers for both X-ray or neutron diffraction studies (Tristram-Nagle, 2007; Sironi et al., 2016). Indeed, they allow lipid multilayers composed of 10–100 stacked bilayers to be produced, which are the ideal samples for neutron diffraction experiments in controlled humidty chambers. We tested and compared these two preparation methods for the production of lipid multilayers with PC-lipids. The collected data suggests that the deposition of lipid vesicle suspension in water allowed for the production of lipid multilayers that better resembled the spontaneous lipid organization in aqueous solution.

**TABLE 1:** Composition of different *P.pastoris* lipid extracts expressed as % mol of the total lipids. The h-phospholipid and d-phospholipid extracts contain all the phospholipids composing the total lipid extract. Their characterization and composition analysis was previously reported (Luchini et al., 2018). In the *E*.*coli* extracts, cyclo C17 and cyclo C19 acyl chains exhibit a cyclopropane ring in their chemical structure.

P.Pastoris Extracts	C16:0	C16:1	C16:2	C16:3	C18:0	C18:1	C18:2	C18:3
*h-phospholipids*	15.9	3.8	1.1	1.9	4.2	41.8	26.4	4.9
*d-phospholipids*	16.7	2.7	0.7	1.3	1.8	72.0	3.6	1.1
*HPC*	10.3	6.3	-	-	20.4	32.1	24.1	6.8
*DPC*	9.4	17.3	-	-	0.9	49.3	19.2	3.8
***E. Coli Extracts***	**C14:0**	**C16:0**	**C16:1**	**C16:2**	**cyclo C17:0**	**C18:0**	**C18:1**	**cyclo C19:0**
*HPG*	5.1	39.7	3.5	2.4	18.7	1.9	6.2	22.4
*DPG*	6.1	45.7	4.1	1.2	25.8	0.9	7.1	8.9

Recently, we also reported a protocol for producing hydrogenous and deuterated lipid extracts from the bacterium *E.coli* (Haertlein et al., 2016). Lipid extracts from *E.Coli* also present a heterogeneous composition of the headgroups and acyl chains. In the present study, we show for the first time the preparation and characterization of the PG lipid extract from *E.Coli*. PG lipids are among the most abundant lipids in the bacterial cytoplasmic membrane. PG lipids have a different acyl chain composition compared to the PC lipids extract from *P.pastoris* and the investigation of the PG multilayer with neutron diffraction allowed us to assess if also in this case lipid self-segregation occurs. As for the PC-lipids, also the PG lipids were produced and characterized in their hydrogenous and deuterated version.

As a result, the hydrogenous and deuterated PC mixtures (hPC and dPC) as well as the hydrogenous and deuterated PG mixtures (dPG and hPG) were characterized by the coexistence of different lipid phases within the multilayer. We hypothesise that, because of the hydrophobic mismatch, the PC and PG lipids with short and long acyl chains tend to separate into different regions of the multilayer, thus originating different sets of diffraction peaks. The PG multilayers exhibited larger *d*-spacing values than the PC multilayers. We propose that such difference is associated both to the different acyl chain composition of the PC and PG mixtures as well as to the electrostatic repulsion between the PG headgroups of consecutive bilayers. Interestingly, both PC and PG extracts showed a slightly different acyl chain composition in the deuterated lipid mixtures compared to the hydrogenous ones. As a consequence, both the dPC and dPG multilayers exhibited some structural differences compared to the hPC and hPG multilayers, respectively. Altogether, we provide valuable information for future application of the hydrogenous and deuterated *P.pastoris* - PC and *E.coli* - PG lipid mixtures, for the development of advanced models of biological membranes and potential drug testing platforms.

## Materials and Methods

### Chemicals

Cell culture reagents were purchased from Sigma Aldrich, France. D_8_-glycerol was from Euriso-Top, France while dio-modified silica column and thin layer chromatography plates (TLC) were purchased from Macherey-Nagel, France. Organic solvents were all HPLC grade, i.e. chloroform (CHCl_3_, ≥ 99.8% purity), ethanol (CH_3_CH_2_OH ≥ 99.8% purity), acetone ((CH_3_)_2_O, ≥ 99.9% purity), methanol (CH_3_OH, ≥ 99.9% purity) (hexane, ≥99.8% purity) and deuterated water (D_2_O, ≥ 99.9% purity) were all purchased from Sigma Aldrich and used without further purification.

### Cell Culture

Hydrogenous and deuterated PC mixtures were extracted from the methylotropic yeast *P.pastoris*, grown either in a hydrogenated or a deuterated culture media, respectively while hydrogenous and deuterated PG mixtures were extracted from *E.coli* cells grown into a hydrogenated and deuterated culture media, respectively. The culture conditions for *P.pastoris* and *E.coli* are extensively described below.

***Pichia pastoris cell culture*** - Cells cultures were carried out as done previously by de Ghellinck et al. (2014), de Ghellinck et al. (2015). Hydrogenated and deuterated *P.pastoris* were cultured in flasks at 30°C using a basal salts medium (BSM) as the minimal medium at pH 6.0 (*Pichia pastoris* fermentation process guidelines, Invitrogen, United States) containing either 20 gL–1 of hydrogenous glycerol in H_2_O or deuterated glycerol (Euriso-Top, France) in D_2_O. Cells upon entering the exponential phase at an OD of 600 were harvested by centrifugation and frozen at −80°C.

***Escherichia coli cell culture*** - Cells were grown on both hydrogenated and deuterated minimal medium at pH 6.0 in flask cultures at 30°C (Haertlein et al., 2016). The composition of the Enfors medium employed was: 6.86 g L-1 (NH_4_)_2_SO_4_, 1.56 g L-1 KH_2_PO_4_, 6.48 g L-1 Na_2_HPO_2_.2H_2_O, 0.49 g L-1 (NH_4_)_2_HC_6_H_5_O_7_ (di-ammonium hydrogen citrate), 0.25 g L-1 MgSO_4_.7H_2_O, with 1.0 ml L-1 of trace metal stock solution (0.5 g L-1CaCl_2_.2H_2_O, 16.7 g L-1 FeCl_3_. 6H_2_O, 0.18 g L-1ZnSO_4_.7H_2_O, 0.16 g L-1CuSO_4_.5H_2_O, 0.15 g L-1MnSO_4_.H_2_O, 0.18 g L-1 CoCl_2_.6H_2_O, 20.1 g L-1 EDTA), 5 g L-1 glycerol. For the preparation of the fully deuterated medium only anhydrous forms of these components were used and diluted into D_2_O. D_8_-glycerol was used as the carbon source. Cells were harvested by centrifugation at their exponential phase (OD 600), and frozen at −80°C.

### Lipid Extraction and Purification

Harvested cells were resuspended into 10 ml deionised water and lysed by probe sonication for 3×5 min with 30 s intervals, 25% duty cycle. The resulting cell lysate was poured into boiling ethanol containing 1% butylated hydroxytoluene (BHT) followed by vigorous stirring in order to denature lipases that have the ability to hydrolyse phospholipids. Lipids were then extracted according to the method of Folch et al. (1957), followed by evaporation of the organic phase under a N_2_ stream and reconstitution of the film was done in CHCl_3_. Purification of various classes of phospholipids containing species of mixed acyl chain lengths was achieved, for both hydrogenated and deuterated mixtures through a diol-modified silica stationary phase column coupled to a High-performance liquid chromatography-Evaporative light scattering detector (HPLC-ELSD) system. The mobile phase employed was a gradient of solvent A (CHCl_3_/MeOH/NH_4_OH, 80:20.5:0.5, v/v) and solvent B (CHCl_3_/MeOH/H_2_O/NH_4_OH, 60:35:5.5:0.5, v/v) (Boselli et al., 2012). TLC analysis was carried out on a High-Performance Thin-Layer Chromatography (HPTLC) system (CAMAG, Muttenz, Switzerland) to assess the identity and purity of each of the purified classes.

### Gas Chromatography

The fatty acid composition of the purified PC and PG natural mixtures was determined by capillary gas chromatography after derivatizing them into their respective fatty acid methyl esters) (FAMEs). About 3 ml of methanolic HCl was added to a glass vial containing 2 mg of purified polar fraction. The vial tube was vortexed, bubbled with Argon, and sealed tightly with a Teflon-lined cap. The solution was incubated at 85°C for 1 h. Once cooled to room temperature (∼10 min), 3 ml of H_2_O was added and the solution vortexed following which 3 ml of hexane was added and vortexed vigorously to create an emulsion. Slow centrifugation at ∼500 g for 5 min at room temperature was carried out to break the emulsion and produce an upper hexane-rich phase containing the FAMEs. This supernatant phase (2.8 ml) was then transferred into a fresh vial, evaporated under a stream of N_2_ and the resulting dried film was reconstituted in 50 μl hexane and transferred into a GC auto sampler vial that was then loaded into the GC’s automatic liquid sampler. The GC instrument (GC 2010 Plus, Shimadzu) was equipped with a split/splitless injector and a SGE BPX70-Cyanopropyl Polysilphenylene-siloxane column (25 m by 0.22 mm ID and 0.25 μm film thickness). Helium was used as a carrier gas at a flow rate of 1.04 ml/min with a linear velocity of 35 cm/s and a purge flow rate of 1 ml/min. The column was allowed to equilibrate for 3 min at 155°C before injection and then the temperature was ramped up to 180°C at a rate of 2°C/min and then to 220°C at a rate of 4°C/min and finally held at 220°C for 5 min resulting in a 27.5 min total run time. Samples (5 μL) were injected into the column at 250°C using an AOC-20i auto injector. Detection was done using a Flame Ionization detector (FID) operating at 260°C with 40 ml/min H_2_, 400 ml/min compressed air and 30 ml/min Helium make-up flow. LabSolutions software (Shimadzu) was used to assign and integrate the total ion chromatogram peaks from which the total mole fraction amounts of individual fatty acids were obtained.

### Lipid Multilayer Preparation

**FIGURE 1:**
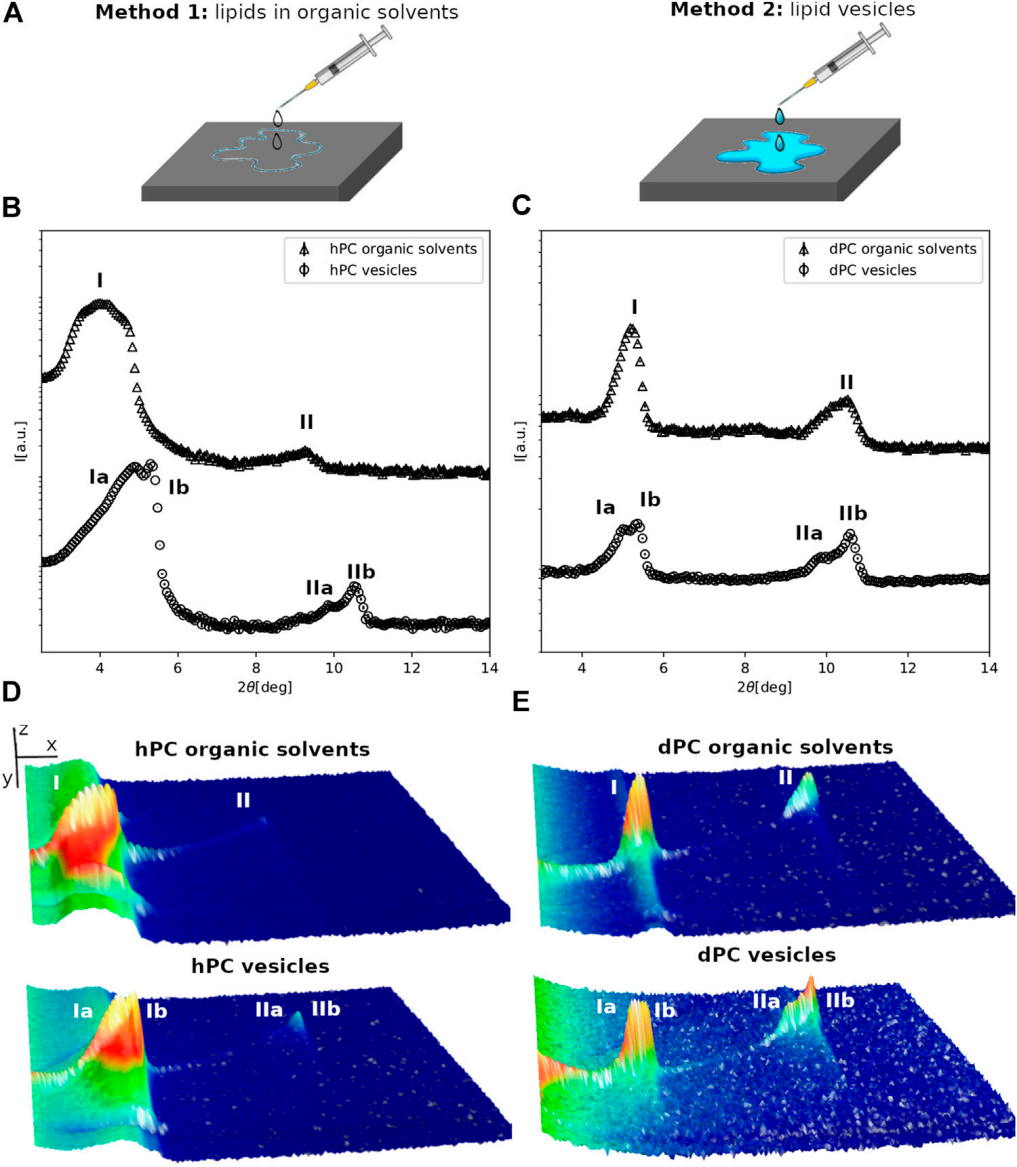
**(A)** Schematic representation of the multilayer preparation methods. **(B,C)** Neutron diffraction data collected for hPC **(A)** and dPC **(B)** multilayers at 57%RH prepared with either method 1, i.e. deposition of lipids dissolved in an organic solvent solution or method 2, i.e. deposition of an aqueous vesicle suspension. **(D,E)** 3D representation of the detector images corresponding to the diffraction profiles reported in **(A,B)**. In **(D,E)** the sample angle (ω) and the angle at the detector (2*θ*) are reported on the *x* and *y* direction respectively, while the intensity of the peak is reported on z direction. In **(B–E)** the different diffraction peaks are identified with Roman numbers, while the letters *a* and *b* are used to distinguish the different lipid phases.

Lipid multilayers were prepared by drying a lipid film on a freshly cleaned quartz support followed by re-hydration in controlled humidity conditions. 50×25×1 mm quartz support were cleaned by sequential sonication in chloroform, acetone, ethanol, ∼15 min each, followed by treatment with UV-ozone lamp for ∼20 min. Lipid multilayers corresponding to 2 mg of hydrogenous or deuterated lipids were prepared with two different methods (Figure 1C). In method 1, a proper amount of a 1:4 chloroform: methanol lipid solution was deposited by drop casting and dried on the cleaned quartz support. In method 2, the same amount of the 1:4 chloroform: methanol lipid solution was first dried on the bottom of a glass vial under nitrogen. Subsequently, the film was re-dissolved with 600 μl of ultra-pure water and sonicated for ∼5 min with a tip sonicator in order to obtain a homogeneous vesicle suspension. The vesicle suspension was deposited by drop casting on a freshly cleaned quartz support and dried. All lipid multilayers, prepared by either method 1 or 2, were stored under vacuum at 45°C for ∼12 h. We also tested shorter annealing time, i.e. 4 h, which did not produce any significant difference in the multilayer structure. The multilayers were subsequently equilibrated at 97% relative humidity (RH) for 24 h and then placed in humidity controlled chamber used as sample environment for the neutron measurements. The reservoir of the humidity chamber was filled with H_2_O for the deuterated multilayers and D_2_O for the hydrogenated multilayers in order to achieve the largest contrast between the lipids and the hydration water (see below).

### Neutron Diffraction

Neutron diffraction data were collected on the cold neutron diffractometer 16D (Cristiglio et al., 2015) at the Institut Laue-Langevin, located in Grenoble, France. Neutrons with 4.5 *Å* wavelength (λ) were produced by reflection of the beam from a highly ordered pyrolytic graphite (HOPG) focusing monochromator. The sample to detector distance was 0.95 m and all samples were measured in reflection mode. The lipid coated quartz supports were mounted vertically on a goniometer head placed in the top compartment of a humidity chamber (Gonthier et al., 2019). The temperature of the sample was maintained at 30.0 ± 0.1°C throughout the measurements while the temperature of the bottom compartment, corresponding to the aqueous reservoir, was adjusted according to the required relative humidity.

Diffraction data were collected at a detector angle (γ) of 12 , by scanning the sample angle (ω) in the range −1 to 10, with a step of 0.05 and acquisition time of 40 s. The neutron scattering intensity was recorded by a position sensitive two-dimensional ^3^He detector (320×320 mm^2^ area with a spatial resolution of (1×1 mm^2^. Samples were first measured at 57% RH and subsequently the RH was increased up to 98% RH. Effective equilibration at 98% RH was monitored by collecting ω-2*θ* scans until no changes were observed in the diffraction pattern. The equilibration at 98% RH required at least 12 h.

Data reduction was carried out with the ILL software LAMP (Large Array Manipulation Program, http:/www.ill.fr/data_treat/lamp/lamp.html). Background subtraction was carried out with a measurement of the empty humidity chamber scattering. The uniformity of the detector efficiency was calibrated with an H_2_O scattering calibration file in LAMP. The reduced 2D images were integrated in the ω range corresponding to the observed diffraction peaks in order to obtain intensity vs 2*θ* plots. The positions of the Bragg peaks in the plots were determined by fitting the peaks with a Gaussian function. The angular position 2*θ* of a Bragg peak is related to the scattering vector (*q*) value by Eq. (1).

$$q=\frac{4\pi \text{sin}\left(\theta \right)}{\lambda }$$ (1)

The diffraction pattern produced by a lamellar phase composed of lipid bilayers alternating with water layers is characterized by Bragg peaks whose *q*-positions correspond to the characteristic ratio *h*/*d*, where *h* is the diffraction order and *d* is the lamellar spacing. Hence, from the *q*_1_ and *q*_2_ position of the first and second order Bragg peaks in the collected data, the characteristic lamellar *d*-spacing can be calculated according to Eq. (2).

$$d=\frac{2\pi }{\left(q_{2}-q_{1}\right)}$$ (2)

The *d*-spacing corresponds to the unit cell thickness (one lipid bilayer and water layer). The errors reported for the *d*-values were obtained by propagation from the uncertainty in the *q*-positions estimated from the fit of the Bragg peaks. Diffraction data collected for samples hydrated with different H_2_O/D_2_O mixtures as well as pure D_2_O and H_2_O can be used to extrapolate the phases of the diffracted waves and hence to calculate the scattering length density distribution across the bilayers. The scattering length density distribution can provide information on how the molecules contained in the sample (in the present case phospholipids) are distributed in the direction perpendicular to the substrate surface. However, this approach can only be applied when a sufficient number of diffraction orders (minimum 3) are observed. Because of the limited number of diffraction peaks produced by the PC and PG multilayers, data were collected in only one contrast (i.e. H_2_O or D_2_O) and this work deals exclusively with the determination of the *d*-spacing. Although a molecular description of the lipid membranes cannot be directly extracted from the collected data, the *d*-spacing evaluation still allowed us the comparison between the overall structure, e.g. membrane thickness or number of lipid phases, of the multilayers prepared with different lipid composition. Further details on the contrast variation method as well as some applications can be found elsewhere (Foglia et al., 2015).

## Results

### Effect of Sample Preparation Method on Multilayer Structure

The hPC and dPC lipid extracts are composed by a single phospholipid headgroup class, i.e. PC, and acyl chains with different length and unsaturation as reported in Table 1 and in Supplementary Material. Figure 1 shows the diffraction data collected for the hPC (1B) and dPC (1C) multilayers at 57% RH. Initially, we characterized the multilayers prepared with method 1 (Figure 1A). As detailed in the materials and method section, the analysis of the diffraction data consists in evaluating the number of Bragg peaks and their relative position. Both in the case of hPC and dPC, two diffraction peaks were detected. In both datasets, the second diffraction order peak, *i.e.* 2*θ ∼* 9 for hPC and 2*θ ∼* 10.6 for dPC, is positioned at twice the 2*θ* value of the first diffraction order peak, *i.e.* 2*θ ∼* 4.5 for hPC and 2*θ ∼* 5.3. Therefore, the 2*θ* spacing between the first and the second diffraction order is compatible with the arrangement of both the hPC and dPC lipids into a lamellar phase. From the 2θ peak positions, and according to Eq. (2), we estimated a *d*-spacing of (49.7 ± 0.3)*Å* for hPC and of (50.4 ± 0.3)*Å* for dPC.

hPC and dPC multilayers prepared with method 2 produced considerably different diffraction profiles, showing additional diffraction peaks compared to the data for the multilayers prepared with method 1. In that case, suspensions of either hPC or dPC vesicles with hydrodynamic radius ∼200 nm, as estimated from dynamic light scattering measurements, were deposited on the quartz support (Figure 1C). Figures 1A,B clearly show that the diffraction profiles are more complex. In fact, based on the 2θ position of the diffraction peaks, both the hPC and dPC multilayers produced two different sets of diffraction peaks suggesting that the multilayers are composed of at least two different lipid phases (named phase *a* and phase *b*). Indeed, the coexistence of different lipid phases characterized by different d-spacing, and therefore producing different sets of diffraction peaks, was already reported for other lipid mixtures produced from *P.pastoris* (Luchini et al., 2018; Luchini et al., 2020) as well as other synthetic lipid mixtures (Mills et al., 2008; Tayebi et al., 2012). The position of the diffraction orders indicates that both phase *a* and *b* are potentially lamellar and the corresponding *d*-spacing values for hPC are *d*_*a*_ = (51.9 ± 0.4)*Å* and *d*_*b*_ = (49.5 ± 0.1)*Å*, while for dPC are *d*_*a*_ = (51.3 ± 0.7)*Å* and *d*_*b*_ = (49.8 ± 0.2)*Å*.

**FIGURE 2:**
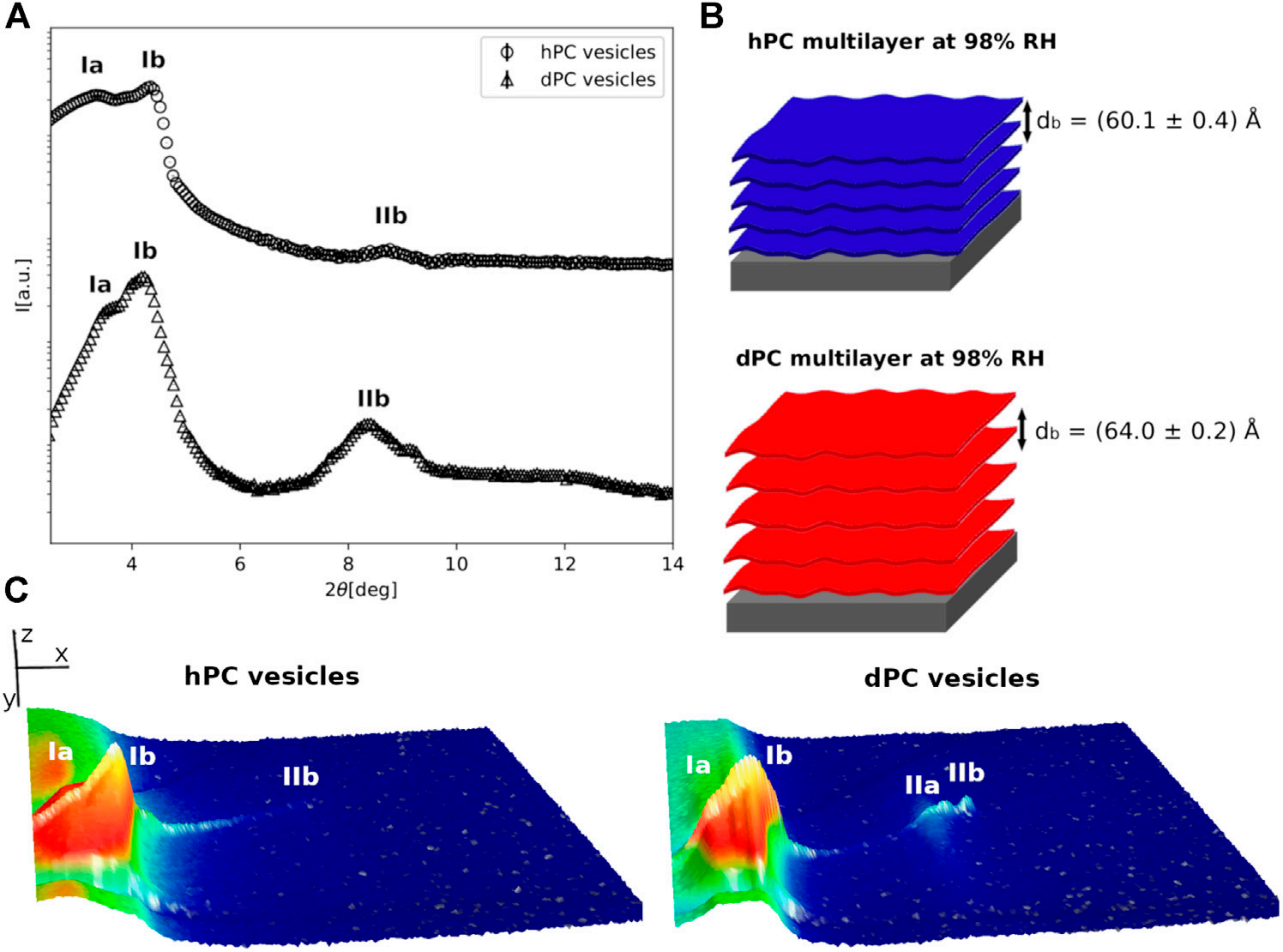
**(A)** Neutron diffraction data collected for hPC and dPC multilayers at 98%RH prepared by deposition of an aqueous vesicle suspension. **(B)** Cartoon representation of the two multilayers with their characteristic *d*-spacing. **(C)** 3D representation of the detector images corresponding to the diffraction profiles reported in **(A)**. In **(C)** the sample angle (ω) and the angle at the detector (2*θ*) are reported on the *x* and *y* direction respectively, while the intensity of the peak is reported on z direction. In **(A**,**C)**, the different diffraction peaks are identified with Roman numbers, while the letters *a* and *b* are used to distinguish the different lipid phases.

Diffraction data were also collected for the multilayers prepared with the two different methods at 98% RH (Figure 2, and SM Figure 1). Indeed, both for hPC and dPC, the multilayers prepared with the two methods exhibited two different sets of diffraction peaks, although not in all datasets two different diffraction orders were resolved.

At 98% RH, in the case of the hPC multilayer prepared with method 1, we were able to identify two lamellar phases with *d*-spacing *d*_*a*_ = (51 ± 1)*Å* and *d*_*b*_ = (53.0 ± 0.1)*Å* (SM-Figure1). The same multilayer prepared with method 2 also produced 2 different sets of diffraction peaks; however, only one lamellar phase (phase *b*) with *d*-spacing *d*_*b*_ = (60.1 ± 0.4)*Å* was identified (Figure 2, SM-Figure 1). Indeed, the other lipid phase (phase *a*) produced a single diffraction peak. Because of the lack of a second diffraction order we were not able to calculate with confidence a corresponding *d*-spacing. Although at 98% RH both method one and 2 produced a similar number of lipid phases within the hPC multilayer, the corresponding *d*-spacing values are considerably different. This indicates that the bilayer thickness and the thickness of the water layers between consecutive bilayers is different in the hPC multilayer prepared with method 1 compared to method 2.

In the case of the dPC multilayers at 98% RH, a lamellar phase with *d*-spacing *d*_*b*_ = (61.8 ± 0.4)*Å* and *d*_*b*_ = (64.0 ± 0.2)*Å* was detected in both the diffraction profiles of the multilayer prepared with method 1 and method 2, respectively (Figure 2, SM-Figure 1). A first diffraction order of an additional lipid phase is also present in the collected data. However, since the peak for the second order is not well-resolved, we were not able to calculate the corresponding *d*-spacing. Interestingly, also in the case of the dPC multilayer, the *d*-spacing calculated for the multilayer prepared by method 1 is smaller than the *d*-spacing calculated for the multilayer prepared by method 2. This observation further supports the hypothesis that the sample preparation method might influence the structure of the produced multilayer.

Altogether, while method 1 produces multilayers with different structure at 57% and 98% RH, a similar arrangement of the multilayers, in terms of number of co-existing lipid phases, is observed at both RH values in case of method 2. The multilayer formation by deposition of the lipid solution in the organic solvents produces multilayers at 57% RH characterized by a single lamellar lipid phase. When the humidity is increased to 98% RH, the fluidity of the bilayers within the multilayers is increased and the lipids can diffuse and eventually self-segregate into domains characterized by a different *d*-spacing. Since all the lipids in the multilayers have the same headgroup, the heterogeneous composition of the acyl chains must drive the observed lipid segregation. In particular, it is reasonable to hypothesise that the PC lipids with shorter acyl chain (*i.e.* C16:0 and C16:1) tend to separate from those with longer acyl chains (*i.e.* C18:0, C18:1 and C18:2) because of hydrophobic mismatch and form slightly thinner bilayer regions within the multilayer compared to the regions formed by lipids with longer acyl chains. Difference in the acyl chain unsaturation level can also favour the lipid self-segregation. As an example, mixtures of DPPC and POPC exhibit the coexistence of fluid and condensed phases (Svetlovics et al., 2012; Åkesson et al., 2012). Indeed in our PC mixtures C18 acyl chains exhibit a higher concentration of single or double unsaturated acyl chains compared to the C16 acyl chains (Table 1). When the multilayers are prepared by deposition of the vesicle suspension, lipids might be already organized into separate domains within the vesicles and are well-hydrated during the formation of the multilayer at the support surface. Therefore, the multilayer appears to be organized into different phases already at 57% RH. We cannot exclude that the observed different structure of the multilayers prepared with method 1 compared to method 2, both in terms of number of lipid phases and the corresponding *d*-spacing values, is also caused by traces of organic solvent molecules among the lipids within the multilayers prepared with method 1.

The collected data suggest that the multilayers prepared with method 2 better resemble the spontaneous organization of the lipids in aqueous solution at the two explored humidity conditions. In addition, method 2 allowed us to mimic physiological conditions during both sample preparation and characterization. Therefore, we decided to adopt method 2 for the comparison of the structure of the yeast and bacterial lipid multilayers.

### Structure of Natural Phosphatidylcholine Multilayers

Figure 2 shows a comparison of the diffraction data collected for the hPC and dPC multilayers at 98% RH. As previously described, at least 2 different lipid phases are present in both multilayers, although for only one of them (phase *b*) two diffraction orders were observed. The 2θ position of these latter suggests a lamellar phase with corresponding characteristic *d*-spacing *d*_*b*_ = (60.1 ± 0.4)*Å* for hPC and *d*_*b*_ = (64.0 ± 0.2)*Å* for dPC. As expected, in both cases, the calculated *d*-spacing resulted to be larger than the corresponding value at 57% RH (Figure 1). Indeed, the increased humidity affects both the lipid organization and therefore the thickness of the bilayers composing the multilayer as well as the amount of water between two consecutive bilayers.

Interestingly, while at 57% RH hPC and dPC multilayers showed a comparable *d*-spacing, at 98% RH the dPC multilayer exhibited a *d*-spacing ∼4*Å* larger than the hPC multilayer. A difference between multilayers prepared with *P.pastoris* hydrogenous and deuterated lipids was already reported in case of multilayers prepared with either the *P.pastoris* total lipid extract or the *P.pastoris* phospholipid extract, both of which contain phospholipids with different headgroups and acyl chains (Luchini et al., 2018; Luchini et al., 2019). In these previous studies, we attributed the different structure of the hydrogenous and deuterated multilayers to the slightly different composition of the headgroups and acyl chains in the hydrogenous and deuterated lipid mixtures. Table 1 shows a comparison of the acyl chain composition analysis for the hydrogenous and deuterated phospholipids, i.e. h-phospholipids and d-phospholipids, which was also previously reported (Luchini et al., 2018), and the one obtained for the hPC and dPC extracts (SM-2). The h-phospholipid and d-phospholipid extract differ from the hPC and dPC extracts both for the composition of the lipid headgroups, different headgroup species are present instead of just PC, as well as for the composition and relative concentration of the lipid acyl chains. In both the d-phospholipid and the dPC extract, the main acyl chain components are C16:0 and C18:1. On the other hand, in the h-phospholipid and the hPC extracts the amount of the different acyl chains is more homogeneously distributed with C16:0, C18:0, C18:1 and C18:2 being the most abundant. Both the phospholipid and PC extracts exhibit different acyl chain composition when produced in their hydrogenous and deuterated versions. However, in the case of h-phospholipid and d-phospholipid extracts the difference in the C18:1 content is more remarkable than in the case of hPC vs. dPC. As a consequence, we reported a difference in *d*-spacing for the h-phospholipid and d-phospholipid multilayers of ∼10*Å* (Luchini et al., 2018), while in the case of the hPC and dPC the difference in *d*-spacing is only ∼4*Å*. Altogether, Table 1 shows that the acyl chain composition is likely to differ depending on the specific lipid headgroup composition.

### Structure of Natural Phosphatidylglycerol Multilayers

**FIGURE 3:**
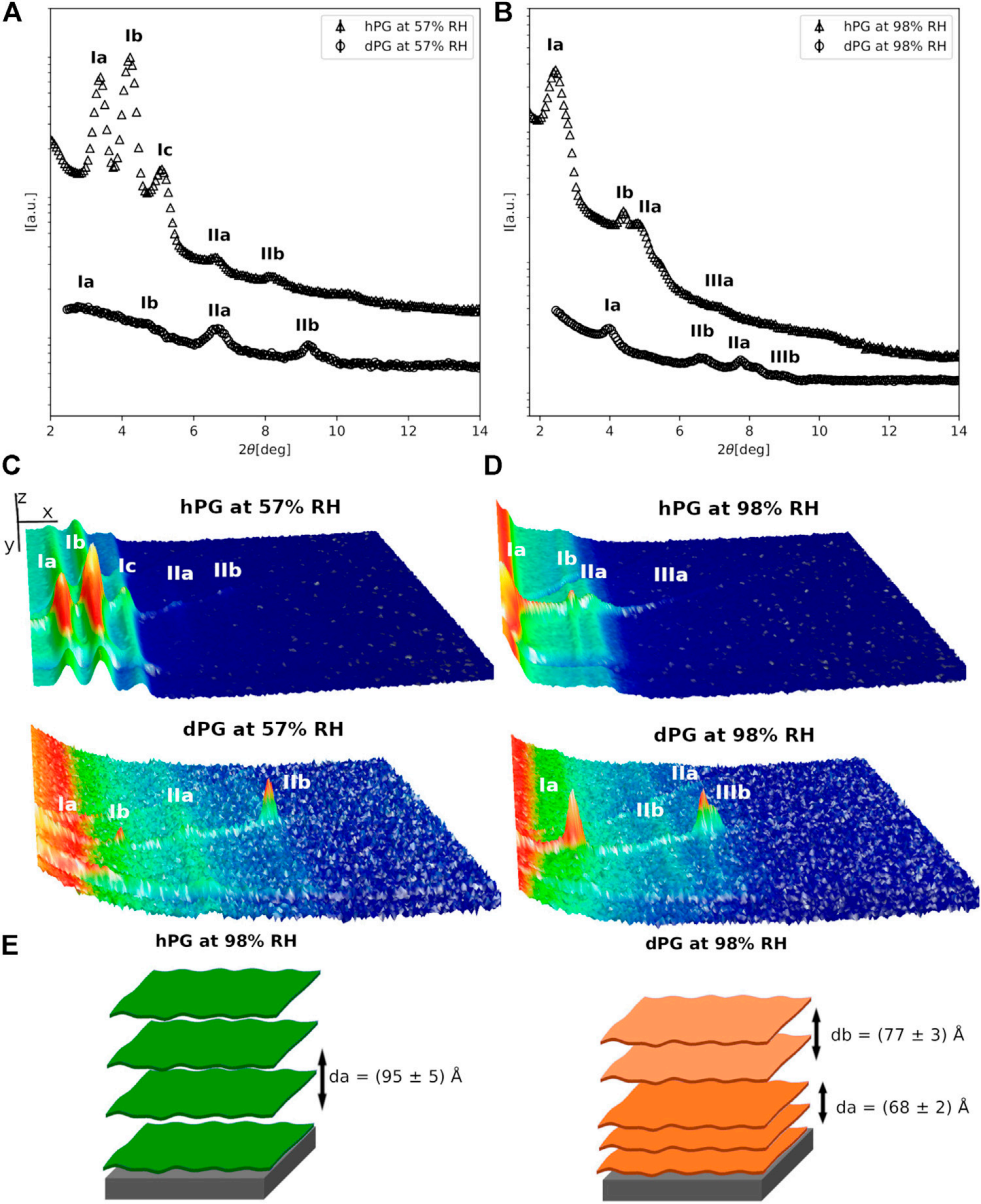
**(A,B)** Neutron diffraction data collected for hPG and dPG multilayers at 57%RH **(A)** and 98%RH **(B)** prepared by deposition of an aqueous vesicle suspension. **(C,D)** 3D representation of the detector images corresponding to the diffraction profiles reported in **(A)**. In **(C,D)** the sample angle (ω) and the angle at the detector (2*θ*) are reported on the *x* and *y* direction respectively, while the intensity of the peak is reported on z direction. In **(A–D)** the different diffraction peaks are identified with Roman numbers, while the letters *a* and *b* are used to distinguish the different lipid phases. The data collected for the hPG multilayer are scaled for visualization. **(E)** Cartoon representation of the two multilayers with their characteristic *d*-spacing.

Figure 3 shows diffraction data collected for the *E.coli* hPG and dPG multilayers at 57% and 98% RH. For both hPG and dPG mixtures, lipid multilayers were prepared by method 2, i.e. suspensions of vesicles with hydrodynamic radius ∼120 nm, as estimated from dynamic light scattering measurements, were deposited on the support. Different diffraction orders belonging to different lipid phases were detected for both the hPG and dPG multilayers at 57%RH. In case of the hPG multilayer, 5 diffraction peaks were detected. By evaluating the position of each of these peaks, we were able to exclude the presence of non-lamellar phases, such as cubic or hexagonal phase, as also expected being the multilayer composed by PG lipids that are known to form lamellar phases (Krumova et al., 2008). Furthermore, the position of the peaks in the 2*θ* range 6–9 *deg* corresponds to twice the 2*θ* position of the first two diffraction peaks at 2*θ∼*3.4 and *∼*4.2. Therefore, we interpreted the diffraction profile as composed by the first and the second diffraction orders of two separate lipid phases named *a* and *b* (Figure 3A). The *d*-spacing associated to the two lipid phases resulted to be *d*_*a*_ = (80.2 ± 0.5)*Å* and *d*_*b*_ = (65.3 ± 0.3)*Å*. An additional diffraction peak is also observed at 2*θ∼*5. This latter most likely belongs to another lipid phase (named *c*), which could not be fully characterized because of the lack of higher order diffraction peaks. At 57% RH, the dPG multilayer also exhibited two different lipid phases. The position of the diffraction peaks suggests two different lamellar phases with *d*-spacing *d*_*a*_ = (76 ± 4)*Å* and *d*_*b*_ = (55 ± 1)*Å*.

When humidity is increased to 98% RH the diffraction peaks collected for both hPG and dPG multilayers showed a lower intensity due to the more disordered structural organization of the multilayers promoted by high humidity. The two lipid phases (i.e. *a* and *b*) in the diffraction data collected at 57%RH, are also observed in both the hPG and dPG multilayers at 98%RH. However, for the hPG multilayer, only in the case of phase *a* two diffraction orders were detectable with associated *d*-spacing *d*_*a*_ = (95 ± 5)*Å*. In the case of the dPG multilayer, the diffraction profile included a sufficient number of diffraction orders to allow us to calculate the *d*-spacing for both lamellar lipid phases *d*_*a*_ = (77 ± 3)*Å* and *d*_*b*_ = (68 ± 2)*Å*. Table 1 shows the acyl chain composition of the hPG and dPG extracts. In both cases, C16:0, cyclo C17:0 and clyclo C19:0 resulted to be the most abundant species. As for the PC mixtures, we hypothesise that acyl chains with the similar length tend to self-segregated into different regions of the multilayer therefore producing at least two observable d-spacings.

Finally, for both hPG and dPG multilayers, increasing the humidity to 98%RH produced a structural rearrangement of the multilayers and more specifically increased the thickness of the two lipid phases observed in the two lipid systems. While at 57%RH the *d*-spacing values estimated for the hPG and dPG multilayer are comparable and within the corresponding errors, at 98%RH the hPG multilayer is evidently characterized by a considerably larger *d*-spacing than the dPG multilayer. The analysis of the acyl chain composition (Table1) indicates that the hPG lipids have a slightly smaller content of short acyl chains (i.e. C14 and C16), but contain double the amount of the long acyl chains (i.e. cyclo C19, see below) compared to the dPG lipids. This difference in the acyl chain composition can contribute to the overall larger *d*-spacing associated to the hPG multilayer compared to the dPG multilayer.

## Discussion

Natural lipids directly extracted from eukaryotic or prokaryotic cells are valuable compounds to produce advanced models of biological membranes. Indeed, they can be characterized by biophysical methods and are pivotal for understanding the physico-chemical properties of real cell membranes as well as to develop lipid platforms for drug-testing. Indeed, more than 60% of the currently marketed drugs target components of cell membranes (Overington et al., 2006). Therefore the development of membrane models close to real biological membranes is of great relevance in the investigation of drug-membrane interactions. A detailed characterization of such interactions can potentially prompt the design of new and more efficient drugs.

In this study, we discussed for the first time the characterization by means of neutron diffraction of hydrogenous and deuterated lipid multilayers prepared with the phosphatidylcholine extract from the yeast *P.pastoris* and the phosphatidylglycerol extract from the bacterium *E.coli*. Specifically, we investigated the impact of sample preparation method and acyl chain composition on the structural organization of the two different kinds of multilayers. The obtained results are summarised in Table 2.

**TABLE 2:** *d*-spacing values calculated for the hPC, dPC and hPG, dPG multilayers at 57% and 98% RH.

***Extracts***	**57% RH**	**98% RH**
*hPC*	*d*_*a*_ = (51.9 ± 0.4); *d*_*b*_ = (49.5 ± 0.1)	*d*_*a*_ = (60.1 ± 0.4)
*dPC*	*d*_*a*_ = (51.3 ± 0.7); *d*_*b*_ = (49.8 ± 0.2)	*d*_*a*_ = (64.0 ± 0.2)
*hPG*	*d*_*a*_ = (76 ± 4); *d*_*b*_ = (55 ± 1)	*d*_*a*_ = (77 ± 3)*d*_*b*_ = (68 ± 2)
*dPG*	*d*_*a*_ = (80.2 ± 0.5); *d*_*b*_ = (65.3 ± 0.3)	*d*_*a*_ = (95 ± 5)

The drop-casting of a lipid solution followed by annealing under vacuum and subsequent re-hydration is one of the common approaches to produce lipid multilayers (Nagle and Tristram-Nagle, 2000; Tristram-Nagle, 2007; Sironi et al., 2016). In the case of the hPC and dPC mixtures, we tested two different sample preparation methods. In method one lipids were dissolved in a chloroform/methanol solution, while in method 2 a lipid vesicle suspension was used. Different multilayer structures were induced by these two methods, similarly to previous findings (Luchini et al., 2020). However, in this previous work, we compared different lipid mixtures prepared with either method 1 or 2. Here, we performed a more systematic characterization and used the exact same lipid mixtures, *i.e.* hPC and dPC, for the comparison of the two sample preparation methods. Altogether, the collected data indicates that the multilayers produced by method 2 show already at 57%RH a structural organization similar to 98%RH, which might be favoured by the spontaneous organization of the lipids into separate domains within the vesicle suspension, compared to the more homogeneous solubilization of the lipids in the organic solvents. In addition, method 2 allowed us to keep the experimental conditions for sample preparation and characterization closer to physiological conditions. Therefore we chose method 2 for the preparation of the samples in the rest of the study.

Both hPC and dPC multilayers exhibited the co-existence of two different lipid phases at both 57% and 98% RH, in case of samples prepared with method 2. Evidence of the coexistence of two lipid phases is the presence of two sets of diffraction peaks in the collected neutron diffraction data, as also previously observed for other lipid mixtures produced from *P.pastoris* (Luchini et al., 2018; Luchini et al., 2020) as well as other synthetic lipid mixtures (Tayebi et al., 2012; Mills et al., 2008). Inspection of Table 1, reporting the acyl chain composition of hPC and dPC extracts, shows that acyl chains are composed by 16 and 18 C atoms and different unsaturations. We suggest that, because of hydrophobic mismatch, the short acyl chains tend to separate from the long acyl chain and form domains within the bilayers, characterized by different *d*-spacing (Table 2). Differences in the acyl chain unsaturation level can also favour lipid self-segregation. Indeed, in both hPC and dPC mixtures, C18 acyl chains exhibit a higher concentration of single or double unsaturations compared to the C16 acyl chains (Table 1). As previously observed for other lipid extracts from *P.pastoris* (Luchini et al., 2018; Luchini et al., 2020), we report differences in the structure of the hPC compared to the dPC multilayers. Such structural differences are related to the different acyl chain composition of the two lipid mixtures and in particular to the higher content of the acyl chain C18:1 in the dPC compared to the hPC.

We also discussed the characterization of lipid multilayers prepared with the bacterial PG extracts produced by *E.coli* cells grown in either a hydrogenous or deuterated culture medium. Interestingly, also in this case the heterogeneity of the lipid acyl chain composition is responsible for the presence of different sets of diffraction peaks belonging to different lamellar lipid phases (Table 2). Indeed, Table 1 shows that acyl chains are mainly saturated and that the most abundant species are C16:0, cyclo C17:0 and clyclo C19:0. As for the PC mixtures, we suggest that acyl chains with similar length tend to self-segregated into different regions of the multilayer therefore producing at least two observable d-spacings. In addition, also the hPG and dPG lipid mixtures exhibited a slightly different acyl chain composition, with C19 acyl chains being more abundant in hPG compared to dPG extracts. This difference in composition is most likely responsible for the larger *d*-spacing measured in the case of hPG multilayers.

Interestingly, at both the two explored humidity conditions, the PG multilayers exhibited a larger *d*-spacing than the PC multilayers. PG lipid extracts have a large content of short acyl chains, *i.e.* C14 and C16 compared to the PC extracts, where the C18 acyl chain is the most abundant (Table 1). However, they also contain a considerable fraction of C19 acyl chains, which are completely absent in the PC extracts. This difference in composition might produce thicker bilayers in the case of the PG extracts compared to the PC. The calculated *d*-spacing depends on both the bilayer thickness and the thickness associated to the water layer separating two consecutive bilayers in the multilayer. Whereas PC lipids have a zwitterionic headgroup, *i.e.* no net charge, the PG lipids are instead negatively charged. Hence, the observed different *d*-spacing in the PG and PC multilayers might also be affected by the electrostatic repulsion between the PG headgroups belonging to two consecutive bilayers, which makes the thickness of the water layers larger in the PG multilayers compared to the PC multilayers.

In conclusion, neutron diffraction allowed us to investigate complex lipid mixtures, such as yeast or bacterial lipid extracts, highlighting their structural organization into different lipid phases. While the co-existence of different lipid phases was previously reported for other yeast lipid mixtures, the current data confirms this behaviour for the case of *P.pastoris* PC and *E.coli* PG multilayers. In particular, we showed that the acyl chain composition of both yeast and bacterial lipid mixtures determines the organization of the multilayers in domains with different *d*-spacing. The novelty of this work resides in the use of natural extracts with a single species headgroup (either PC or PG) and a mixture of chain compositions coming from yeast or bacterial cells. These lipids assemble in lamellar structures of great bio-relevance while the possibility to use fully deuterated material allows a fine characterisation by neutron scattering.

## Data Availability

The dataset presented in this study are stored in the online repository of Institut Laue Langevin (data@ill.eu).
