# Supplementary material for: Structural Characterization of Natural Yeast Phosphatidylcholine and Bacterial Phosphatidylglycerol Lipid Multilayers by Neutron Diffraction
Source: Front Chem. 2021 Mar 18;9:628186. doi: 10.3389/fchem.2021.628186 (PMC8104085; doi:10.3389/fchem.2021.628186)
Supplement: Supplementary file 1 [file datasheet1.pdf]

Supplementary Material

1 SUPPLEMENTARY FIGURES

1.1 Effect of the sample preparation method on the hPC and dPC multilayer structure

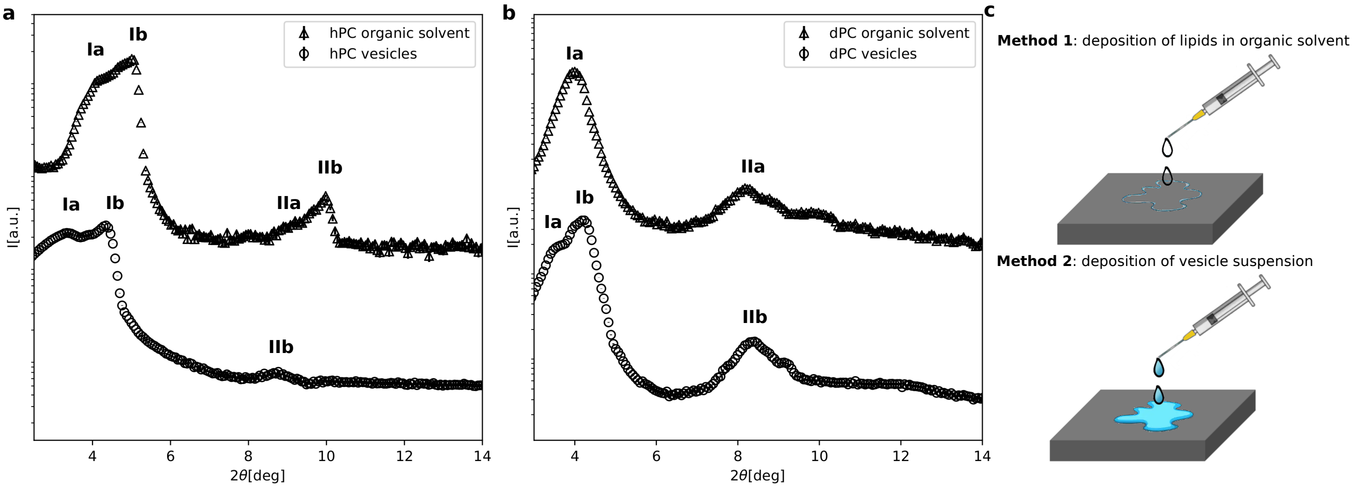

Figure S1. Neutron diffraction data collected for the hPC (a) and dPC (b) multilayers at 98%RH prepared with either method 1, i.e. deposition of the lipids in an organic solvent solution or method 2, i.e. deposition of the aqueous vesicle suspension. In (a) and (b) the different diffraction peaks are identified with roman numbers, while the letters *a* and *b* are used to distinguish the different lipid phases. (c) cartoon representation of the two sample preparation methods

1.2 Acyl chain composition hPC and dPC extracts

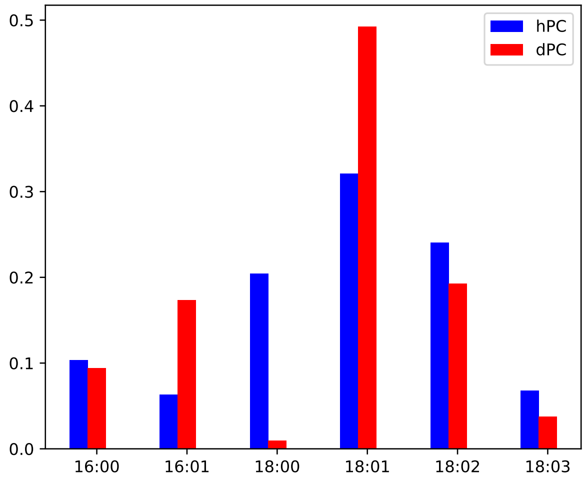

Figure S2. Gas chromatography -mass spectrometry (GC-MS) analysis of the acyl chain composition of the hPC and dPC extracts

1.3 Acyl chain composition hPG and dPG extracts

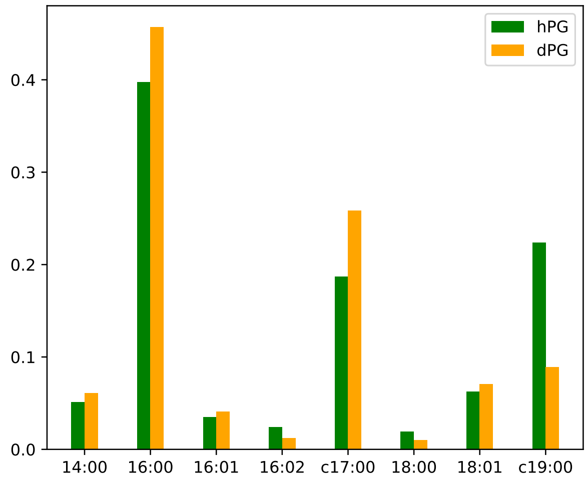

Figure S3. Gas chromatography -mass spectrometry (GC-MS) analysis of the acyl chain composition of the hPG and dPG extracts. c17:00 and c19:00 are the PG acyl chains exhibiting a cyclopropane ring.
